# Essentials of Physics and Chemistry

**Published:** 1886-04

**Authors:** 


The Essentials of Physics and Chemistry. *By* Condict W. Cutler, m. d. *New York:* J. H. Vail & Co.

This is a small book, or, better, a collection of "pocket editions" published at different times, and now bound together. The whole is a fair illustration of the frauds offered to medical students, and is characterized by careless composition, incorrect statements and a complete misconception of what a medical or general student should know of physics and chemistry.

